# Postpartum Plasma-Derived Exosomes Confer Mitochondrial Stabilization and Neuroprotection against Ischemic Stroke

**DOI:** 10.21203/rs.3.rs-9573067/v1

**Published:** 2026-05-27

**Authors:** Xiaoyun Sun, Lijun Xu, Majesty Greer, Elijah McMillan, Oiva Arvola, Pervez Sultan, Kazuo Ando, Jiong-Jin Min, Vishal Chavda, Julian Wolf, Vinit Mahajan, Brendan Carvalho, Creed M. Stary

**Affiliations:** Stanford University School of Medicine; Stanford University School of Medicine; Howard University; Howard University; University of Helsinki; Stanford University School of Medicine; Stanford University School of Medicine; Samsung Medical Center, Sungkyunkwan University School of Medicine; Stanford University School of Medicine; Molecular Surgery Laboratory, Stanford University School of Medicine; Molecular Surgery Laboratory, Stanford University School of Medicine; Stanford University School of Medicine; Stanford University School of Medicine

**Keywords:** Heat shock protein, mitochondrial dynamics, neuroprotection, astrocytes, transcriptomics

## Abstract

**Background:**

The molecular mechanisms governing adaptive neuroprotection during the postpartum period remain unknown. We hypothesized that circulating exosomes contain bioactive cargo (such as Hsp20) that confer neuroprotection against ischemic injury during the postpartum period.

**Methods:**

Exosomes were isolated from plasma of postpartum female mice (ppExos) and control female mice, and from serial blood samples obtained from healthy human volunteers during pregnancy (3rd trimester) and again on postpartum day 2. Exosomal size and protein markers were confirmed *via* nanoparticle tracking analysis and Western blotting. Neuroprotection with exosome treatment was assessed *in vitro* using mouse neuronal and astrocyte cultures and human retinal pigment cell line subjected to simulated ischemia. I*n vivo* neuroprotection was assessed using transient middle cerebral artery occlusion (MCAO) in young adult and aged mice. Mitochondrial integrity and reactive oxygen species (ROS) production were evaluated by live cell imaging. *In vivo* neuroprotection was evaluated by assessing infarct volume and neurobehavioral scores. Changes in mitochondrial dynamics were measured by immunofluorescence and Western blot. Human exosomes were sent for proteomic assessment (SomaScan^™^) followed by differential expression analysis (R software v4). Protein quantification was validated by immunoblot.

**Results:**

ppExos significantly reduced infarct volumes and improved neurological deficits post-MCAO in both young and aged female mice. *In vitro*, ppExos reduced ROS generation in all cell types after simulated ischemia and reduced mitochondrial fragmentation in astrocytes. Mitochondrial fusion proteins Mfn2 and Opa1 proteins were elevated in maternal postpartum brains, and preserved in both astrocyte and neuronal cell cultures after *in vitro* ischemia with ppExo treatment. Proteomics revealed significant upregulation of heat shock protein 20 (Hsp20) in human ppExos, while Western blot validated elevated Hsp20 in both human and mouse ppExos. Simulated ischemia significantly reduced Hsp20 in astrocyte and neuronal cultures which was reversed by treatment with ppExos. In conclusion, ppExos represent a previously unrecognized, naturally optimized neuroprotective agent that enhances mitochondrial resilience and antioxidant defenses associated with enhanced Hsp20 expression. These findings establish a novel platform for sex-informed, cell-free therapies in ischemic cerebrovascular accidents.

## INTRODUCTION

Ischemic cerebrovascular accidents (CVA) is a leading cause of adult disability and the second leading cause of death worldwide [1]. Women in the peripartum period represent a well-established population at elevated CVA risk, with childbirth and the acute postpartum period representing a select population at up to 3-fold higher risk of ischemic CVA [2, 3]. One widely recognized barrier to translational success in the development of novel therapies and understanding mechanisms for neuroprotection against peripartum stroke is the long-standing utilization of male-only rodent models of experimental CVA. Evidence for this is substantiated by known sex differences in stroke outcomes [1]. The elevated CVA risk in the immediate postpartum period has been attributed to normal physiologic hemodynamic changes that occur during childbirth and to a shift from an anticoagulant to a procoagulant state to achieve hemostasis [4]. Notably, embolic and thrombotic events are most frequent in the early postpartum window and largely resolve after 6 weeks postpartum. Despite a well-established increase in stroke risk during the immediate peripartum period that is driven by hypercoagulability, endothelial dysfunction, and cardiopulmonary stress, the molecular and cellular mechanisms that determine injury severity and recovery remain poorly defined.

Paradoxically, our prior work in mice [5] demonstrates that the postpartum state confers robust neuroprotection following experimental cerebral ischemia. This apparent disconnect between increased stroke incidence and reduced injury severity suggests the activation of endogenous, compensatory protective programs during the peripartum period. This suggests an evolutionary strategy whereby compensatory neuroprotective mechanisms have evolved during the peripartum period to reduce injury if a CVA occurs. We hypothesize that the peripartum period could represent an evolutionary adaptation that offsets the elevated risk of CVA associated with normal physiological changes of pregnancy and childbirth. We propose that these adaptations have evolved to buffer the maternal brain against the heightened vascular risk associated with pregnancy and childbirth.

Over the prior decade, extracellular vesicles, particularly exosomes, have emerged as promising candidates for CVA therapy [6, 7]. Exosomes are nano-sized vesicles (30–150 nm) secreted by nearly all cell types that carry a diverse cargo of bioactive molecules, including proteins, lipids, and RNAs, capable of modulating recipient cell behavior [8, 9]. Importantly, they can cross the blood-brain barrier [10], exhibit low immunogenicity, and have demonstrated therapeutic efficacy in a range of neurological disease models [11, 12], including CVA. Several studies have shown that exosomes derived from mesenchymal stem cells (MSCs) or other cells can reduce infarct size, modulate inflammation, and promote neurogenesis in experimental stroke models [13] [14]. However, little is known about the influence of physiological states, such as pregnancy and the postpartum period, on the neuroprotective potential of circulating exosomes. Evidence suggests that exosomal cargo is highly dynamic and responsive to physiological changes [8, 9]. For example, breast milk exosomes from lactating females have been shown to contain elevated levels of immunomodulatory miRNAs known to modulate inflammation, promote cell survival, and influence neural development [15]. Exosomes isolated from the plasma of postpartum females may similarly be enriched in neuroprotective factors. Unlike artificially engineered vesicles, naturally occurring exosomes represent a biologically tuned system shaped by evolutionarily conserved maternal adaptations [15].

Therefore, to resolve the paradox between the clinical vulnerability of the peripartum state and experimental evidence of biological resilience against CVA, we tested the novel hypothesis that postpartum plasma-derived exosomes (ppExos) confer neuroprotection against experimentally induced CVA, potentially involving enhanced mitochondrial resilience, anti-inflammatory signaling, and stress-response modulation. Unveiling the mechanisms by which this population is protected opens a new translational avenue for identifying circulating and transferable cell-free therapies that can be extended beyond maternal health and benefit the broader stroke population.

## MATERIALS and METHODS

Animal Models and Ethics Statement: All animal experiments were conducted in accordance with the National Institutes of Health guidelines for the care and use of laboratory animals and were approved by the Stanford University Institutional Animal Care and Use Committee (APLAC #A3213–01). Female C57BL/6 mice (8–12 weeks old) were bred and observed for delivery. Postpartum mice were defined as those within 2–3 days post-delivery. Age-matched nulliparous (control) C57BL/6 females, age-matched male and aged female (16–18 months) C57BL/6 mice were used for comparison studies. For human plasma samples, IRB approval (#51414) and consent was obtained prior to phlebotomy and biobanking.

Exosome Isolation and Characterization: After sacrifice, whole blood was extracted from non-pregnant and postpartum mice. Serial human whole blood samples were obtained from three healthy women volunteers at two time points: once in their 3rd trimester (> 28 weeks gestational age) prior to onset of labor, and again on postpartum day 2 following scheduled cesarean delivery. Exosome extraction from human and mouse plasma was performed as previously described [16]. Briefly, cell culture supernatant was centrifuged at 300 g for 10 min and passed through 0.2 um pore bottle top filter (BD Falcon, #352340, Thermo Fisher Scientific). One quarter volume of PEG (polyethylene glycol 8000, Thermo Fisher Scientific) stock solution [(20 g PEG8000 in 20 ml 0.5 NaCl phosphate-buffered saline (PBS, Sigma)] was added. Next, the total volume was brought to 50 ml with deionized H_2_O and incubated overnight at 4°C. Samples were centrifuged at 1500 g for 30 min, then at 300 g for 5 min after removal of most supernatant. The final pellet was then resuspended in PBS. Protein concentrations were determined *via* the bicinchoninic acid method (BCA Protein Assay Kit; Pierce). The Malvern Panalytical NanoSight^™^ instrument was used to confirm size distribution (30–150 nm). Western blotting was used to confirm exosome isolation using the exosomal marker CD63 (#315108, Abcam).

Transient Middle Cerebral Artery Occlusion (MCAO): Focal cerebral ischemia was produced by 1h of transient middle cerebral artery occlusion (MCAO) using a silicone-coated 6 − 0 monofilament (Doccol, Sharon, MA) as previous [16, 17]. Separate animals underwent sham surgery (anesthesia and incision but no MCAO). As sex and age play a critical role in stroke outcomes [1], we assessed the effect of ppExos in protection against experimental CVA in young adult (8–10-week old) and aged (18–20-month old) females, and in young adult males. Temperature and respiratory rate were monitored continuously. The rectal temperature was maintained at 37 ± 0.5°C controlled by a homeothermic blanket control unit (Harvard Apparatus). Mice were randomly assigned to receive intravenous exosome treatment (50 μg in 100 μl saline) or saline administration alone *via* jugular vein injection immediately prior to emergence from anesthesia [17].

Determination of Infarct Volume and Neurological Status: Cerebral infarction volume was determined at 1d post-MCAO with 2,3,5 triphenyltetrazolium chloride (TTC, Sigma, T8877) after transcardiac perfusion with saline then fixation with 4% paraformaldehyde[16, 17]. Infarct volume was quantified using four 50 μm coronal sections/brain and corrected for edema as previously described [16, 17]. Neurological status was assessed after 1 day of reperfusion using a 4-point neurologic deficit score as previously described [16, 17]. Neurological deficit was scored as follows: 0 - no observable neurological deficits, 1 - failure to extend right forepaw, 2 - circling to the right, 3 - falling to the right, 4 - cannot walk spontaneously [16, 17].

Brain Fluorescent Immunohistochemistry: Animals were sacrificed at 24 h after MCAO by isoflurane overdose, and brains immediately perfused with transcardial ice-cold saline, then fixed with 4% phosphate-buffered paraformaldehyde (PFA) for stereological analysis. Coronal vibratome sections (50 μm) were used for fluorescent immunohistochemical (IHC) analysis as previous [18]. Fixed sections were stained for the astrocyte marker glial fibrillary acidic protein (GFAP, #ab90601, Abcam), the mature neuronal marker NeuN (#ab104224,) and the mitochondrial fusion proteins mitofusin-1 (Mfn2, #ab124773, Abcam) and OPA1 (#ab157457, Abcam), followed by secondary antibody solutions (Alexa Fluor 350nm, 488nm, and 594nm, Invitrogen) in PBS at 4°C, overnight [18]. Images were acquired by an observer blinded to conditions using an upright Zeiss Axio-Imager M2 fluorescent microscope equipped with Apotome 2.0 for optical sectioning, and Zeiss EC-Plan Neofluar 20X, Zeiss Plan Apochromat 40X, and Zeiss LC-Plan Neofluar 63X objectives [18]. All imaging for a given protein were collected using a fixed excitation intensity, exposure time, and gain, to minimize variability. An observer blinded to conditions quantified the relative intensity of Mfn2 and OPA1 from maximum projection Z-stack images using StereoInvestigator^™^ (MicroBrightField) and ImageJ v1.49b (NIH, Bethesda, MD), as we have previously reported [18].

Cell Cultures and Simulated Ischemia: *Primary cortical astrocytes* were prepared from postnatal (days 1–3) mouse pups as previously described [16]. Briefly, following euthanasia with isoflurane, neonatal brains were isolated and cortices were microdissected and dissociated with 0.05% trypsin (ThermoFisher Scientific) in DMEM medium (ThermoFisher Scientific) for 20 min. Following trypsinization, cells were dissociated by trituration and then resuspended in DMEM medium supplemented with 10% FBS and 1% penicillin/streptomycin. The culture medium was exchanged every two days and the cells were used for experiments after > 70% confluence at 16–18 days in vitro (DIV). *Primary neuronal cultures* were prepared as previously described [16] from embryonic (E16–E18) mouse cortices. Briefly, the dissected cortices were dissociated with 0.05% trypsin/EDTA for 15 min at 37°C, triturated, then plated in medium containing 5% FBS and 5% ES (HyClone). A relatively pure neuronal culture was obtained by adding cytosine arabinoside (3 mol/liter, Sigma). Immortalized human epithelial cells originally sourced from ATCC were obtained as a gift from the laboratory of Dr. Jeffrey Goldberg (Stanford University, Dept. of Ophthalmology, Stanford, CA). Cells (25–33 passage) were seeded on 24-well plates at 1.5 × 10^5^ density in plating medium consisting of Eagle’s Minimal Essential Medium (Gibco), supplemented with 10% fetal bovine serum (Hyclone) as previous [19]. Cultures were maintained at 37°C in a 5% CO_2_ incubator until confluent then subjected to 1 hour (neurons) or 5 hours (astrocytes) oxygen-glucose deprivation (OGD) injury using an anoxia chamber (Coy), or to 8 hours glucose deprivation injury (GD, ARPE-19 cells).

Live-cell Fluorescent Imaging: *Reactive oxygen species production* was performed as previous [20]. After treatment with exosomes and OGD, oxygen radical production was assessed in astrocyte cultures using hydroethidine (HEt, ThermoFisher Scientific). Cultures were incubated in the dark with 5 μM HEt in BSS5.5 (30min, 37°C), and the same concentration of HEt was maintained in the bath throughout each experiment. Cultures were imaged using an automated LumaScope^™^ 720 (Etaluma) with a 20X fluor objective. Cells were maintained at 37°C and 5% CO_2_ in an atmospherically controlled imaging chamber (Ibidi GmbH). Mean fluorescence intensity was quantified using ImageJ v1.49b (NIH). *Mitochondrial morphology* was assessed in astrocytes as previous [21]. Primary astrocyte cultures were incubated in MitoTracker^™^ Green FM stain (ThermoFisher Scientific) at 200 nM in PBS solution for 30 min. Next, the cells were washed one time with a culture medium and incubated with SiR-actin stain (Cytoskeleton, Inc.) solution at 0.5 μM for an additional 3 h. Finally, Hoechst 33528 dye (ThermoFisher Scientific) was added to the cell culture medium at 1 μg/ml and incubated for 10 min followed by imaging with a Zeiss Observer inverted fluorescence microscope at 400 × magnification. For analysis of mitochondrial morphology, the mitochondrial network analysis (MiNA) ImageJ macro tool was employed [22] to determine the percentage of mitochondrial fragments (< 300 mm^2^), as previously described [21].

Cell Culture Fluorescent Immunohistochemistry: *In situ* semi-quantitative protein imaging was performed *via* fluorescent immunocytochemistry as previous [18, 21]. Cells were washed once with 0.1% PBS, fixed with ice-cold 4% paraformaldehyde for 10 min and then washed again three times with PBS. Afterward, the cells were permeabilized with 0.1% Triton-X in PBS for 10 min followed by blocking with 5% horse serum (HyClone) for 1 hour. Primary antibodies against Mfn2 (Abcam #124773) and OPA1 (Abcam #157457) were diluted in blocking buffer and added to cells and then incubated overnight at 4°C for 24 hours. Next, cells were washed three times with PBS, followed by the addition of secondary antibodies (1:10000) and the nuclear dye DAPI (4′,6-diamidino-2-phenylindole, ThermoFisher Scientific). Cells were imaged with a Zeiss Observer inverted fluorescence microscope at 400 × magnification by an observer blinded to conditions [18, 21].

Protein Quantification: Isolated human exosomes were sent for proteomic assessment to Somalogic using the SomaScan^™^ 7K DNA-adaptomer platform. Astrocyte culture total protein was isolated as previous [18, 21]. Briefly, cultures were washed once with cold 0.1% PBS then harvested and lysed in RIPA buffer with phosphatase inhibitor cocktail-2 (Sigma-Aldrich). Total cellular protein was quantified by Pierce BCA protein assay kit (Thermo Fisher Scientific), as described previously [18, 21]. Equal amounts of protein were loaded and separated on 10–12.5% polyacrylamide gels, then transferred to Immobilon polyvinylidene fluoride membrane. Membranes were blocked and incubated overnight with primary antibody against Hsp20 (HSPB6, Abcam, #ab184181) and the exosome indicator CD63 (Abcam, #ab217345). Next, membranes were washed and incubated with secondary antibodies for 1 h followed by washing again and visualizing by using the LICOR Odyssey infrared imaging system [18, 21].

Densitometric analysis of bands was performed via Image Studio Lite (LI-COR Biosciences), and the intensity of all proteins was normalized to beta-tubulin as a control [18, 21].

Statistical Analyses: Data were analyzed using Graphpad Prism Software; ANOVA with Tukey’s post hoc test or Student’s t-test. A p-value < 0.05 was considered statistically significant. Proteomic differential expression analysis was performed using R software (v4).

All *in vivo* experiments were performed with n ≥ 6 animals, and all *in vitro* experiments were performed in triplicate.

## RESULTS

### Plasma-derived postpartum exosomes provide neuroprotection in vivo.

**Figure 1:**
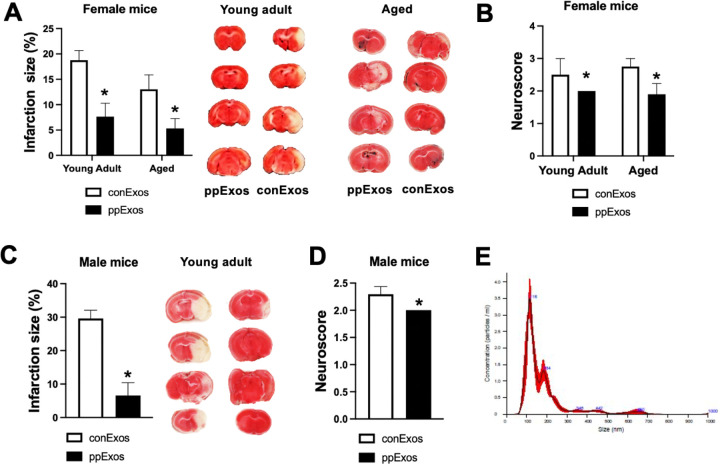
Postpartum mice demonstrate protection against transient middle cerebral artery occlusion (MCAO) and its recapitulation by circulating exosomes. **(A)** Infarct volume (left) and representative TTC-stained brain sections (right) in non-pregnant young adult females and aged females 24 hours after MCAO. Cohorts were treated 2h post-MCAO with either exosomes derived from separate cohorts of control (non-pregnant) females (“conExos”) or postpartum females (“ppExos”). **(B)** Quantification of neurological deficit in the same females. **(C)** Infarct volume (left) and TTC-stained brain sections (right) in young adult male mice 24 hours after MCAO, treated 2h post-MCAO with either conExos or ppExos. **(D)** Quantification of neurological deficit in the same males. **(E)** Nanosite^™^ quantification of particle size validating circulating postpartum extracellular vesicles as exosomes. N=6–10, *= p<0.5 versus conExos.

To test the hypothesis that circulating exosomes from postpartum females are, at least in part, responsible for protection against experimental stroke during the postpartum period, we isolated exosomes from the plasma of postpartum (ppExos) and control (conExos) female mice and then administered them to naïve (non-pregnant) female mice 2 hours after MCAO. In parallel, we included a cohort of aged females to assess the biological variable of lifespan. In both young adult female mice and in aged female mice, ppExos treatment resulted in significantly (p < 0.05) reduced infarct volumes compared to conExos-treated mice (Fig. 1A). Improved neuroprotection in both female cohorts with ppExos compared to conExos was confirmed by improved neurobehavioral performance (Fig. 1B). The neuroprotective effect of ppExos also extended to young adult male mice, underscored by both a reduction in infarct volume and an improvement in neurobehavioral outcomes (Fig. 1C,D). These results demonstrate that ppExos are not only sufficient to recapitulate the neuroprotective effect of the postpartum state in age-matched young adult females but are also broadly effective in reducing ischemic brain injury across the lifespan, and independent of sex [9, 23, 24. Nanosite particle tracking was used to confirm the average particle size, with the majority corresponding to < 200 nm (Fig. 1E), the size that defines exosomes {Robbins, 2016 #254].

### Postpartum exosomes suppress oxidative stress and preserve mitochondrial morphology.

**Figure 2:**
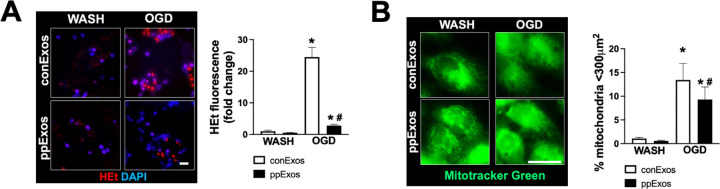
Postpartum derived exosomes exert antioxidant effects and preserve mitochondrial ultrastructure in mouse astrocytes after *in vitro* ischemia. **A)** Example images (left) and quantification (right) of the reactive oxygen species indicator hydroethidine (HEt, red) and the nuclear die DAPI (blue) in mouse astrocyte cultures subjected to 5 hour oxygen-glucose deprivation (OGD) incubated in either conExos or ppExos. **B)** Example images (left) and quantification (right) of the mitochondrial fragmentation using Mitotracker Green^™^ in mouse astrocyte cultures subjected to 5 hour oxygen-glucose deprivation (OGD) incubated in either conExos or ppExos. N=6, *= p<0.5 versus sham, #=p<0.05 versus female control. Scale bar = 15 μm.

To further explore mechanisms underlying ppExo-mediated protection, we performed several experiments to evaluate their effect on mitochondrial function. We first assessed whether ppExos could play a protective role by preventing the generation of ROS in primary mouse astrocyte cultures. Cells were first pre-treated with either ppExos or conExos and then immediately exposed to 5 hours of oxygen-glucose deprivation (OGD) injury. Fluorescent ROS imaging revealed that OGD triggered a substantial increase in superoxide production in conExos-treated cultures, which was markedly attenuated by ppExos treatment (Fig. 2A). These findings suggest that ppExos likely mitigate ischemia-induced oxidative injury through modulation of redox homeostasis. Given the central role of mitochondrial dysfunction in ischemic injury, we then assessed the impact of ppExos on mitochondrial architecture with the same injury and treatment paradigm in astrocyte cultures. Mitochondrial morphology assays revealed that OGD caused prominent mitochondrial fragmentation in astrocytes, characterized by short, punctate morphologies (Fig. 2B). In contrast, ppExo-treated cells maintained elongated and interconnected mitochondrial networks indicative of healthy dynamics (Fig. 2B).

#### The postpartum state and ppExos preserve mitochondrial dynamics

**Figure 3:**
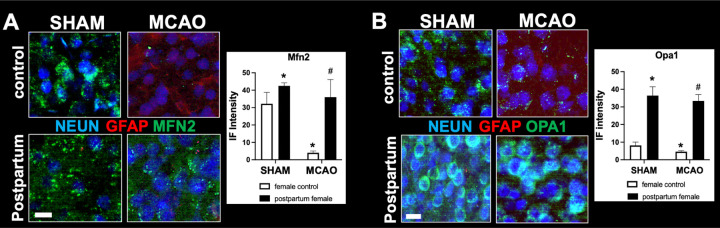
Postpartum brains express higher levels of mitochondrial dynamic proteins at baseline and in response to MCAO. **A)**Example images of control (non-pregnant) and postpartum mouse cortex 6h after SHAM surgery or middle cerebral artery occlusion (MCAO). Sections (left) stained with the neuronal marker NeuN (red), the astrocyte marker glial fibrillary acidic protein (GFAP, red), and the mitochondrial fusion protein Mfn2 (green); unbiased* quantification of MFN2 fluorescence intensity is on the right. **B)** Sections (left) of cortex stained with NeuN, GFAP, and the mitochondrial fusion OPA1 (green); unbiased quantification of OPA1 fluorescence intensity is on the right. *All images represent identical excitation light intensity, exposure duration, and image gain and brightness setting. N=6, *= p<0.5 versus sham, #=p<0.05 versus female control. Scale bar = 15 mm.

**Figure 4:**
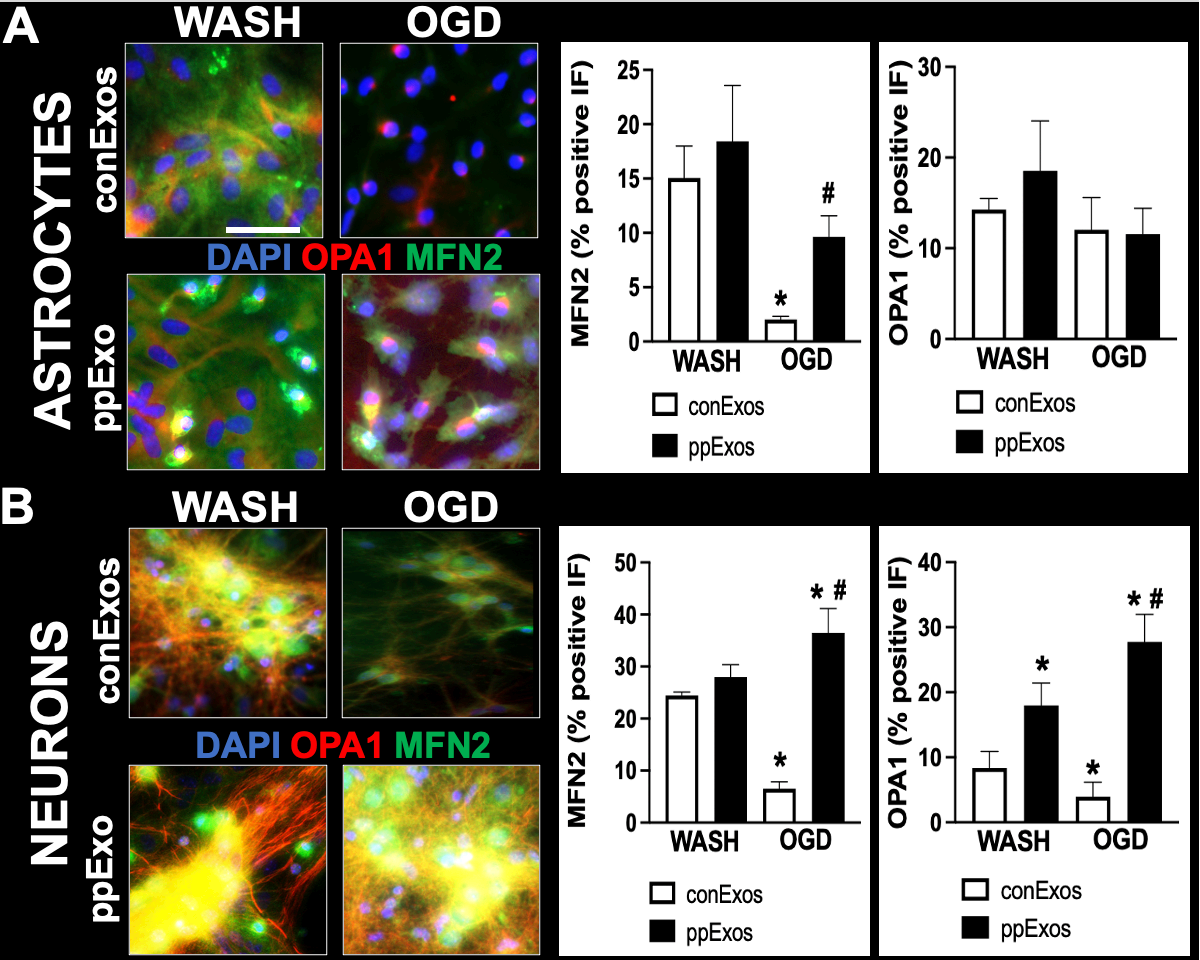
Astrocyte and neuronal cell cultures express higher levels of mitochondrial dynamic proteins at baseline and in response to simulated ischemia after incubation with ppExos. **A)**Example images of mouse cortical astrocyte cultures following 5 hours of oxygen-glucose deprivation (OGD) treatment or control wash, and incubation with either conExos or ppExos. Cultures (left) stained are the nuclear dye DAPI (blue), and the mitochondrial fusion proteins OPA1 (red) and Mfn2 (green), with quantification of MFN2 and OPA fluorescence on the right. **B)** Examples of mouse cortical neuronal cultures following 5 hours of OGD or control wash, and incubation with either conExos or ppExos, stained with DAPI (blue), OPA1 (red) and Mfn2 (green), with quantification of MFN2 and OPA fluorescence on the right. N=6, *= p<0.5 versus conExo wash treatment, #=p<0.05 versus conExo OGD treatment. Scale bar = 25 mm.

Preservation of mitochondrial morphology was further supported by *in vivo* immunostaining. Cortical sections from MCAO mice treated with ppExos displayed increased expression of mitochondrial fusion proteins Mitofusin-2 (Mfn2) and OPA1 in both neurons and astrocytes, as visualized *via* immunofluorescence (Fig. 3). Quantitative analysis revealed significantly higher fluorescent signal intensities in the ppExo group compared to controls (Fig. 3), supporting a role for ppExos in stabilizing mitochondrial fusion machinery *in vivo* (29)(30)(31). We extended these findings by analyzing the cell-type specific effects of ppExos on Mfn2 and OPA1 expression in primary cortical neurons and astrocytes following OGD. Both cell types showed marked preservation of Mfn2 and OPA1 in the presence of ppExos (Fig. 4), indicating a cell-type-independent effect of mitochondrial protection via maintaining dynamic stability. Conversely, conExo-treated cultures exhibited severe depletion of these proteins (Fig. 4). Together, these data support a model in which ppExos prevent global mitochondrial fragmentation and maintain fusion-fission balance under ischemic stress across multiple cell types. As neuronal and astrocytic metabolic homeostasis and ATP production are maintained *via* distinct pathways, these results suggest that ppExos act to preserve bioenergetic integrity through a common modality [25–28].

### Human ppExos are protective in vitro and in vivo

**Figure 5:**
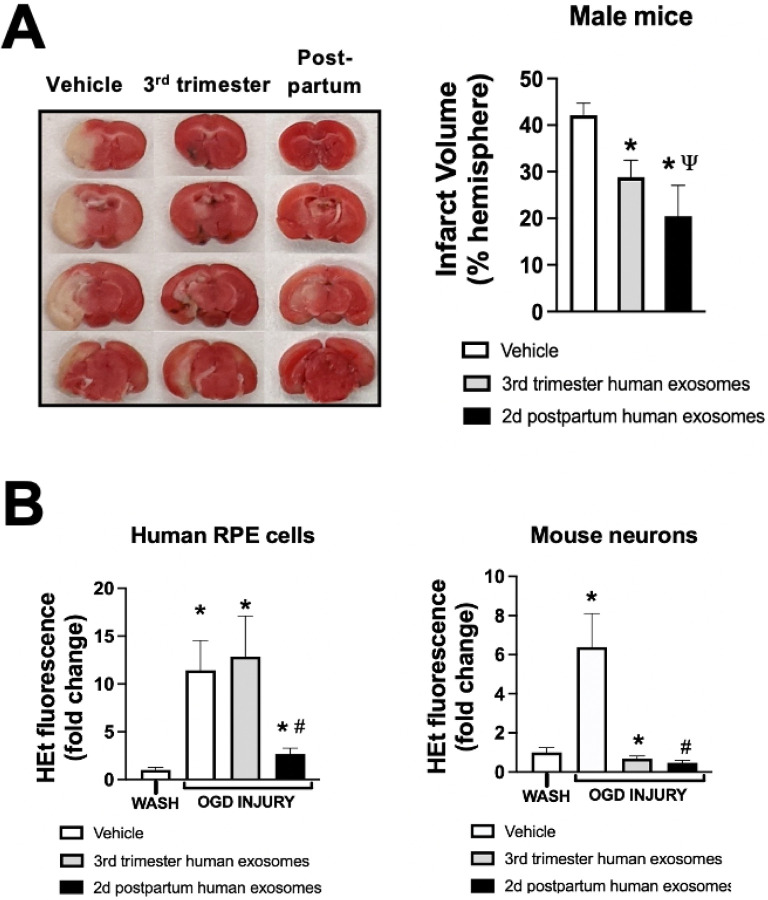
Human pregnancy (3^rd^ trimester) and postpartum-derived [BC1] exosomes recapitulate protection from ppExos. **(A)** Reactive oxygen species generation (Hydroethidine, Het, fluorescence) 12 hours after simulated ischemia (oxygen-glucose deprivation, OGD) in human retinal pigment epithelial (RPE, left) or in mouse neuronal cultures (right) after incubation with either saline (vehicle), exosomes isolated from the plasma of women in their 3^rd^ trimester of pregnancy versus or after incubation with exosomes isolated from the same women sampled 2 days postpartum. **(B)** TTC stained brain sections (left) 24h after MCAO (viable tissue in red) and quantification of infarct volume (right) in male mice treated intravenously 2h post-MCAO with either exosomes derived from women in their 3^rd^ trimester of pregnancy, or with exosomes derived from the same women 2 days postpartum. N=6, *= p<0.5 versus control or wash or vehicle control, #=p<0.05 versus stem cell exosomes. [BC1]Some is 3^rd^ trimester

To assess translational relevance, we next interrogated the protective role of human postpartum exosomes. Plasma was first collected longitudinally from three different peripartum women at two time points: the late 3rd trimester and the postpartum period (within 48 hours post-delivery). Exosomes were purified and administered to adult male mice intravenously 2 hours after MCAO. Intravenous administration of both human 3rd trimester and postpartum exosomes to adult male mice resulted in significant reductions in infarct volumes (Fig. 5A), highlighting the therapeutic efficacy of peripartum bioactive molecules across species and experimental models. However, human ppExos exhibited an enhanced protective benefit compared with human 3rd trimester exosomes. We next assessed whether a similar mitochondrial-protective effect of human ppExos could be elicited across species and cell types. In human epithelial cells, human ppExos, but not 3rd trimester exosomes, significantly reduced ROS production, as visualized by hydroethidine (HEt) fluorescence (Fig. 5B). Interestingly, in mouse neuronal cell cultures, both human ppExos, and human 3rd trimester exosomes exhibited strong antioxidant properties. These results demonstrate that human exosomes isolated from women in the peripartum period carry cargo that is strongly neuroprotective across different cell types and animal species.

#### Heat shock protein 20 is enriched in ppExos from both mice and humans

**Figure 6:**
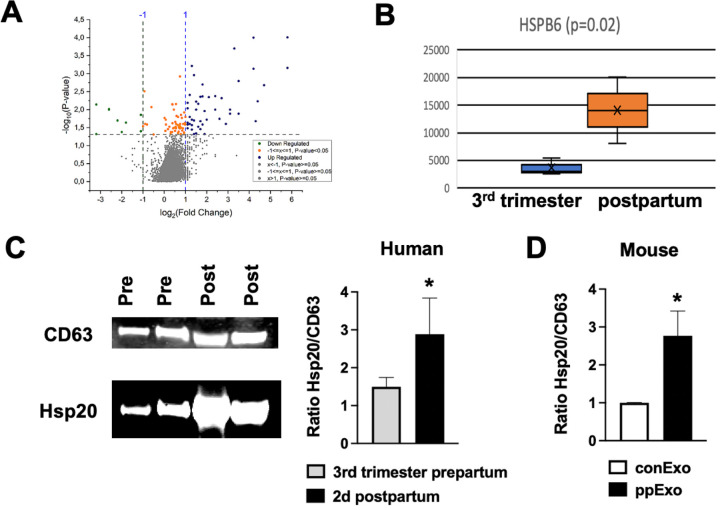
Elevated Hsp20 expression in human and mouse ppExos. **(A)** Volcano plot results of proteomic chip analysis illustrating differentially expressed proteins in human prepartum and postpartum exosome samples. **(B)** HspB6 (Hsp20) is identified as significantly (p<0.05) higher expressed. **(C)** Immunoblot of Hsp20 and the exosome marker CD63 from prepartum (pre) and postpartum (post) exosome samples (left) and quantification of the ratio of Hsp20/CD63 in exosome samples. **(D)** Immunoblot quantification of the ratio of Hsp20/CD63 in mouse conExo and ppExo samples. N=3 per group, *p<0.05.

**Figure 7:**
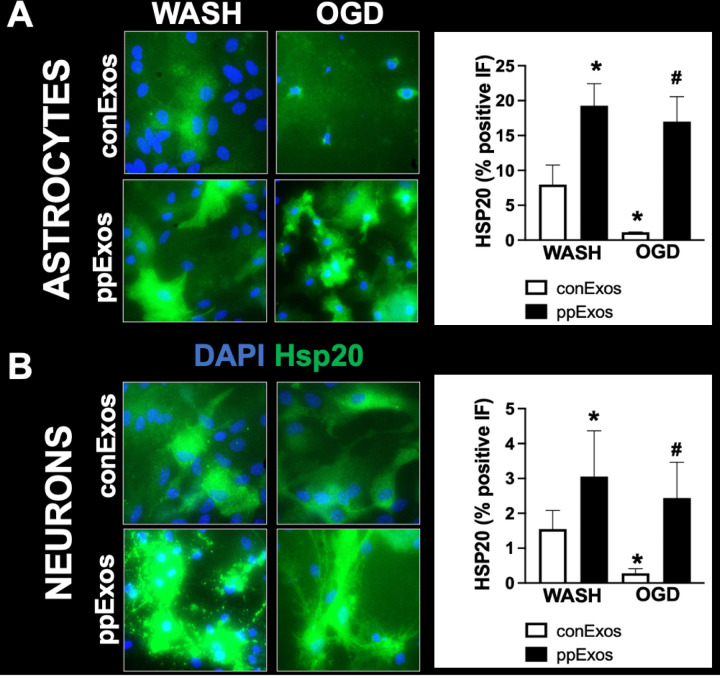
Astrocyte and neuronal cell cultures express higher levels of Hsp20 at baseline and in response to simulated ischemia after incubation with ppExos. Example images of mouse astrocyte cultures **(A)** and mouse neuronal cultures **(B)** following incubation with either conExos or ppExos and OGD. Cells are stained with DAPI (blue) and Hsp20 (green). Quantification of Hsp20 fluorescence for each cell type is on the right. N=6, *= p<0.5 versus conExo wash treatment, #=p<0.05 versus conExo OGD treatment. Scale bar = 25 mm.

In order to identify potential neuroprotective cargoes carried within ppExos we performed proteomic analyses (Fig. 6A). Exosomal cargo from both mouse and human plasma identified Heat Shock Protein 20 (Hsp20, also known as HSPB6) as one of the most differentially expressed proteins in ppExos (Fig. 6B). Western blotting confirmed > 3-fold elevation of Hsp20 in ppExos (Figs. 6C,D). Given the known roles of Hsp20 in cytoprotection, mitochondrial stability, and redox regulation [29–31], we subsequently evaluated Hsp20 expression in cultured neurons and astrocytes treated with conExos or ppExos under OGD conditions. Hsp20 measurements in astrocyte cell cultures (Fig. 7A) revealed a significant decrease in Hsp20 expression with conExo treatment, which was attenuated with ppExo treatment. A similar increase was observed in neuronal cultures, (Fig. 7B) suggesting that Hsp20 expression may play a role in the protective effect of ppExos [29–31].

## DISCUSSION

Pregnancy and the postpartum period is marked by numerous physiological, immunological, and metabolic changes designed to support pregnancy maintenance, fetal development, maternal recovery and maternal-infant bonding [4]. The postpartum period is also associated with an increased risk of CVA, attributed to fluctuations in coagulation profiles, endothelial function, and systemic hemodynamics [4]. However, our prior preclinical work demonstrates that the postpartum state confers significant neuroprotection in a murine model of ischemic CVA compared to age-matched nulliparous females [5]. Interestingly, prior work by Ritzel *et al*. demonstrated that multiparity in mice improved outcomes after experimental stroke despite features of increased CVA risk [32]. Moreover, our prior study in mice [5] and two clinical studies [33, 34] have identified nursing during the postpartum period as an associated behavior that confers significant neuroprotection against maternal CVA. Collectively, these findings support the hypothesis that despite an elevated risk of maternal CVA during the peri-partum period, maternal physiologic changes simultaneously confer a degree of neuroprotection should a CVA occur.

In the present study, we observed that the neuroprotective phenotype of the maternal postpartum brain against experimental stroke could be recapitulated by administration of exosomes derived from postpartum mice (ppExos). The protective effect was consistent in non-pregnant female mice, in aged female mice, and in male mice, demonstrating that ppExos were broadly effective across biological variables (Fig. 1). This sex and age independence is particularly important given the high burden of CVA in older adults and the unique pathophysiology of CVA in older women. Our findings suggest that ppExos could help close the translational gap by providing consistent therapeutic benefit across these variables. Moreover, human ppExos also provided neuroprotection against experimental stroke in mice (Fig. 5), demonstrating that ppExos also bridge species differences that have traditionally limited the translational success of novel stroke therapies [25, 35]. This novel finding identifies a translational opportunity that may be particularly relevant in high-risk populations with elevated stroke incidence, including patients with hypertensive disorders of pregnancy such as preeclampsia, cardiomyopathy, or thrombophilia, where absolute ischemic stroke risk may increase several-fold to an estimated 30–150 per 100,000 deliveries (16484606; 35477033). While direct clinical translation of postpartum plasma is not yet feasible due to the need for mechanistic definition, standardization, and safety validation, these data provide a strong rationale for developing targeted, biologically derived therapies and for future early-phase trials in carefully selected high-risk peripartum populations or in patients presenting with acute stroke.

Mitochondrial dysfunction plays a central role in ischemic injury, leading to energy failure, oxidative stress, and activation of cell death pathways [13]. Recent work has demonstrated that exosomes can be employed to preserve mitochondrial function in different recipient cell types [26, 27]. In the present study, we observed that mouse ppExos demonstrated robust protection against *in vitro* ischemia in mouse astrocyte cultures (Fig. 2), associated with a reduction in ROS production, and maintenance of mitochondrial morphology. A similar reduction in ROS was observed in human RPE cells following administration of human ppExos (Fig. 5). Notably, human ppExos were also protective in mouse astrocytes (Fig. 5), further validating the cross-species relevance of the postpartum protective phenotype. These findings suggest that physiological events surrounding childbirth may alter the content and function of circulating exosomes in ways that enhance mitochondrial function in recipient cells, to ultimately impart therapeutic properties.

Mitochondrial dynamics encompass the coordinated processes of fusion, fission, transport, and turnover. Together, these processes sustain bioenergetic efficiency and overall mitochondrial function [28]. Fusion allows mitochondria to share metabolites, proteins and mitochondrial DNA, supporting oxidative phosphorylation and buffering local damage. Disruption of mitochondrial dynamics impairs ATP generation, increases oxidative stress, and compromises cellular resilience, underscoring their central role in sustaining metabolic health and cell survival [28]. Our *in vivo* findings revealed for the first time that the postpartum state is associated with enhanced expression of the key mitochondrial fusion proteins Mfn2 and OPA1 (Fig. 3). Maintenance of these proteins are essential for maintaining mitochondrial integrity, bioenergetic efficiency, and resistance to apoptotic stimuli following ischemia [36, 37]. Administration of ppExos *in vitro* reproduced this effect by preserving mitochondrial fusion proteins in both astrocyte and neuronal cultures (Fig. 4). Collectively, our findings suggest that the mitochondrial-stabilizing effects of ppExos may coordinate their neuroprotective capacity and occur independently of cell type.

Next, to elucidate any bioactive components responsible for the protective effects of ppExos, we conducted a comparative proteomic analysis of exosomes isolated from plasma collected from women during the 3rd trimester of pregnancy and from the same patients within 48 hours after childbirth. We observed significant enrichment of Hsp20 in ppExos versus patient-matched 3rd trimester exosomes (Fig. 6). Hsp20 is a small heat shock protein known for its role in cytoprotection, where it has been shown to modulate mitochondrial function, inhibit apoptosis, and attenuate oxidative stress [29–31]. We validated Hsp20 enrichment in both mouse and human ppExos (Fig. 6) and we observed that Hsp20 expression decreased in both astrocyte and neuronal cultures after in vitro ischemia (Fig. 7). Notably, treatment with ppExos preserved Hsp20 levels after injury in both cell types across all mouse groups (Fig. 7). These results suggest that Hsp20 could be a critical effector molecule within ppExos, contributing to observed neuroprotection and modulation of mitochondrial homeostasis and redox balance.

### Limitations.

While our findings are robust and promising, some limitations should be acknowledged. The murine and human exosome studies were not directly matched in design. In mice, exosomes were obtained from separate non-pregnant and postpartum animals, while in humans, samples were collected longitudinally from the same individuals during the third trimester and again after delivery, with both time points showing protective effects. We selected the 3rd trimester to represent maximal pregnancy-associated changes, however physiological and biological differences are dynamic during pregnancy. Although significant differences were identified in the protective effect between pregnant and ppExos, the sample size was small and additional validation studies are necessary. We demonstrated the presence and function of Hsp20 in both human and mouse exosomes, however we did not perform comprehensive loss-of-function studies for all other candidate proteins or RNAs. Finally, the long-term effects of ppExos on neuroplasticity, angiogenesis, and cognitive recovery remain to be determined in future studies.

### Conclusions.

This study identifies peripartum plasma exosomes (ppExos) as potent, naturally occurring neuroprotective agents in ischemic CVA. We demonstrate that ppExos confer significant protection against experimental stroke and preserve mitochondrial integrity across sex and age cohorts. Importantly, human third-trimester exosomes were also protective, indicating that induction of cytoprotective mechanisms are established at least during late pregnancy. Mechanistically, protection may be mediated, at least in part, by the antioxidant and mitochondrial stabilizing functions of Hsp20, which is enriched in ppExos. These findings position postpartum exosomes as a biologically tuned, sex-informed, and cell-free therapeutic platform with high translational potential for ischemic CVA. The clinical implications of our findings are significant. Exosomes can be isolated from human blood plasma, scaled for clinical use, and administered intravenously. In parallel, exosomes provide an enriched biological derivative containing the key bioactive mediators of plasma-derived protection, enabling both direct therapeutic use and systematic identification of the specific proteins, lipids, and RNAs that could be engineered or manufactured as defined next-generation therapeutics. Unlike live cell therapies, exosomes pose a lower risk of immunogenicity, tumor formation, or vascular occlusion [13]. Furthermore, the postpartum period may serve as a model for other condition-specific states of enhanced repair. However, because it is also associated with a heightened risk of venous thromboembolism, a leading cause of maternal mortality, future studies must determine whether postpartum-derived exosomes exert protective, neutral, or prothrombotic effects on coagulation and vascular function before therapeutic translation. Future efforts should focus on identifying the cellular origin of ppExos and determining whether their production can be induced or engineered in non-postpartum donors.

## Supplementary Material

Supplementary Files

This is a list of supplementary files associated with this preprint. Click to download.

- FigureS1.docx
